# Sinisan ameliorates colonic injury induced by water immersion restraint stress by enhancing intestinal barrier function and the gut microbiota structure

**DOI:** 10.1080/13880209.2023.2191643

**Published:** 2023-04-04

**Authors:** Xiaoying Xu, Huimei Hu, Haizhou Zeng, Boyi Li, Qiuxiong Yin, Yupeng Jiang, Linquan Zang, Changlin Zhao, Guoqiang Qian

**Affiliations:** a School of Chinese Medicine, Guangdong Pharmaceutical University, Guangzhou, P.R. China; b College of Pharmacy, Guangdong Pharmaceutical University, Guangzhou, P.R. China; c Health Science College, Guangdong Pharmaceutical University, Guangzhou, P.R. China

**Keywords:** Inflammation, intestinal flora, tight junction, intestinal barrier, brain-gut peptide, microbiome-gut-brain axis

## Abstract

**Context:**

Sinisan (SNS) has been used to treat psychosomatic diseases of the digestive system. But little is known about how SNS affects water immersion restraint stress (WIRS).

**Objective:**

To study the effects of SNS on colonic tissue injury in the WIRS model.

**Materials and methods:**

Forty-eight Kunming (KM) mice were randomized into 6 groups (*n* = 8): The control and WIRS groups receiving deionized water; the SNS low-dose (SL, 3.12 g/kg/d), SNS middle-dose (SM, 6.24 g/kg/d), SNS high-dose (SH, 12.48 g/kg/d), and diazepam (DZ, 5 mg/kg/d) groups; each with two daily administrations for 5 consecutive days. The 5 treatment groups were subjected to WIRS for 24 h on day 6. The effects of SNS on colon tissue injury caused by WIRS were assessed by changes in colon histology, inflammatory cytokines, brain-gut peptides, and tight junction (TJ) proteins levels. 16S rRNA gene sequencing was used to detect the regulation of the gut microbiota.

**Results:**

SNS pretreatment significantly reduced TNF-α (0.75- to 0.81-fold), IL-6 (0.77-fold), and IFN-γ (0.69-fold) levels; and increased TJ proteins levels, such as ZO-1 (4.06- to 5.27-fold), claudin-1 (3.33- to 5.14-fold), and occludin (6.46- to 11.82-fold). However, there was no significant difference between the levels of substance P (SP) and vasoactive intestinal peptide (VIP) in the control and WIRS groups. SNS regulated the composition of gut microbiota in WIRS mice.

**Conclusion:**

The positive effects of SNS on WIRS could provide a theoretical basis to treat stress-related gastrointestinal disorders.

## Introduction

Stress is a serious threat with short-term and long-term effects on gastrointestinal function (Bhatia and Tandon 2005). Acute restraint or immobilization stress in rodents is usually used to investigate the effect of stress on intestinal function. There are generally three types of stress models, including restraint stress (RS), cold restraint stress (CRS), and WIRS models, all of them include both physiological and psychological stressors (Söderholm and Perdue 2001). Among various gastrointestinal disorders, the effects of physical and psychological stress are widely recognized (Li et al. 2011). Some stressful life events are associated with the onset or symptom exacerbation of common gastrointestinal diseases, such as peptic ulcer and inflammatory bowel disease (IBD) (Mayer 2000). The WIRS model is commonly used for stress-induced gastric ulcers (Zhang et al. 2022). It has also been found that acute WIRS leads to duodenal injury (Xu et al. 2018) and impaired colonic mucosal function (Li et al. 2011). However, the pathogenesis of colonic injury in the WIRS model is still unclear and needs to be further investigated.

Tight junction (TJ) proteins are significant parts of the mucosal barrier (Martini et al. 2017; Tan et al. 2019). The transmembrane proteins, occludins and claudins, components of TJ proteins, interact to form a physical barrier between adjacent epithelial cells. The coprotein zonula occludens (ZO) family is a cytoplasmic protein that connects TJ components to the cytoskeleton (WEN et al. 2014; Assimakopoulos et al. 2020). The expression of occludin and ZO-1, markers of epithelial tight junctions in gastric tissue, was significantly reduced after 16 h of WIRS in rats (Huang et al. 2020). We hypothesize that WIRS may damage the integrity of the colon barrier in mice.

The gut microbiome is a vital component of the microbiome-gut-brain axis (MGBA), which can be affected by stress (Bear et al. 2021). SP and VIP are the excitatory and inhibitory neuropeptides of the gastrointestinal tract. Patients with IBD have lower VIP and higher SP levels in their colonic mucosa (Patel et al. 2020). Gut bacteria live in symbiosis with intestinal cells. Intestinal symbionts support intestinal barrier function, medication metabolism, food metabolism, and avoidance of harmful microbial invasion (Jandhyala et al. 2015). Imbalances between intestinal commensal and pathogenic floras can lead to dysregulation of the gut flora (Nishida et al. 2018). Psychological stress aggravates inflammation by changing intestinal permeability to allow harmful bacterial products to pass through intestinal epithelium (Hills et al. 2019).

It has been proven that SNS has many effects, including antidepressant (Shen et al. 2020), anti-inflammatory, regulating intestinal flora (Zhu et al. 2019), and improving experimental colitis in mice (Sun et al. 2009; Cai et al. 2021). SNS also can counteract psychological stress and stress-related disorders caused by chronic restraint stress in rats (Cheng et al. 2017). SNS is a typical formula in Chinese medicine for treating disorders related to psychological stress, including depression (Feng et al. 2016), anxiety (Tanaka et al. 2013), gastroesophageal reflux disease, peptic ulcer, irritable bowel syndrome (IBS), ulcerative colitis (UC) and functional dyspepsia (Ling et al. 2015). We established the WIRS model and verified it. By observing the histopathological changes of the colon, inflammatory factors, TJ proteins, brain-gut peptides and intestinal flora, we hope to uncover the protective effects of SNS on the colon tissue in WIRS mice and provide the reference and basis for further research.

## Materials and methods

### Preparation of SNS

SNS was prepared from the following ingredients: *Bupleurum chinense* DC. (Apiaceae), *Paeonia lactiflora* Pall. (Paeoniaceae), *Citrus aurantium* L. (Aurantioideae), and *Glycyrrhiza uralensis* Fisch. (Fabaceae), with a dose proportion of 1:1:1:1 (shown in Table 1). Our team obtained and verified the herbs from Beijing Tongrentang (Guangzhou, China). Four Chinese medicinal herbs (12 g each) were soaked for 1 h and then decocted twice for 1 h each. Two filtrates were combined and concentrated to 1 g/mL. The SNS solution was stored in a refrigerator at 4 °C. By diluting 1 g/mL SNS with ultrapure water, the low-, medium-, and high-dose concentrations of SNS were 0156, 0.324, and 0.624 g/mL, respectively. The medium dose was considered to be the therapeutic comparable dose for humans. Reference standards of saikosaponin A (CAS No. 20736-09-8, purity ≥ 98%), paeoniflorin (CAS No. 23180-57-6, purity ≥ 98%), naringin (CAS No. 10236-47-2, purity ≥ 98%), and glycyrrhizic acid (CAS No. 1405-86-3, purity ≥ 98%) were all purchased from Shanghai Macklin Biochemical Co., Ltd. (Shanghai, China). Diazepam tablets were manufactured by Shandong Xinyi Pharmaceutical Co., Ltd. (Lot number: NO.210504, national medicine permission number: H37023039). Diazepam was diluted with ultrapure water to a concentration of 0.25 mg/mL. The clinical adult dose recommended in the medication instructions served as the basis for the diazepam dose used in our study.

**Table 1.** Crude drug composition of SNS.

Plant name	Chinese name	Place of origin	Voucher number	Major components
*Bupleurum chinense* DC.	Chaihu	Hebei, China	21020507	Saikosaponin A
*Paeonia lactiflora* Pall.	Baishao	Anhui, China	21050201	Paeoniflorin
*Citrus aurantium* L.	Zhishi	Jiangxi, China	20210104	Naringin
*Glycyrrhiza uralensis* Fisch. ex. DC.	Gancao	Inner Mongolia, China	21101604	Glycyrrhizic acid

### Determination of SNS

High-performance liquid chromatography (HPLC) was used for the SNS quality control by an Agilent Technologies 1260 Infinity system with a 1260 DAD VL detector. A C18 column (Thermo Acclaim120, 4.6 mm × 250 mm, 5 mm) was used for the analysis, and the column temperature was maintained at 30 °C. The mobile phase was composed of water containing 0.1% phosphoric acid (A) and acetonitrile (B). The linear gradient elution program for the mobile phase was as follows: 0–35 min, 5–-26% B; 35–80 min, 2650% B; 80–90 min, 50–65% B; 90–105 min, 65–85% B; 105–110 min, 85–5% B. At 210 nm, monitoring was carried out. The injection volume was 10 μL.

### Animals

Forty-eight healthy male Kunming mice (age 7–8 weeks old, body weight 20–22 g) were obtained from the Experimental Animal Management Center of Southern Medical University (Guangzhou, China). Animals were given water and food while being kept in a specific pathogen-free laboratory with a 12 h light/dark cycle. Before experiments, mice needed to acclimated for 5 days. The Guangdong Pharmaceutical University’s Animal Ethics Committee approved all experimental procedures (approval number: gdpulac2022116), which were carried out under the National Institutes of Health Guidelines for Laboratory Animals.

### Animal groups and drug administration

Forty-eight KM mice were randomly assigned to 6 groups (*n* = 8 per group): the control and WIRS groups receiving deionized water; the SNS low-dose group (SL group), SNS middle-dose group (SM group), SNS high-dose group (SH group) receiving 3.12, 6.24, and 12.48 g/kg/d SNS, respectively; and the diazepam group (DZ group) receiving diazepam at 5 mg/kg/d. Each group received two daily intragastric doses for 5 days.

### Water immersion restraint stress

The 5 treatment mouse groups were subjected to water immersion restraint stress modeling for 24 h starting on day 6. The modeling scheme was modified from a previous report (Zhang et al. 2021). The mice (except their heads) were completely immersed in water at 21 ± 2 °C for 24 h, while they were restrained on the wire mesh to prevent them from moving. The water level was maintained at the level of the sternal process of the mice.

### Sample collection

At the end of the modeling, all mice were sacrificed by cervical dislocation. Brain tissue samples were collected immediately by craniotomy on a sterile operating platform. Subsequently, the entire intestine was taken from the pylorus to the end of the colon, and the feces and cecum contents were collected for 16S rRNA gene sequencing. The intestinal wall was washed with saline, then photographed and stored in 4% paraformaldehyde or cryopreservation tubes. The feces, brain and colon tissue samples in the cryopreservation tubes were immediately immersed in liquid nitrogen for 1 min and held at −80 °C for subsequent experiments.

### Hematoxylin–eosinstaining

The colonic tissue was fixed for 24** **h in 4% paraformaldehyde, embedded in paraffin wax, cut into 5** **μm sections, and stained with Hematoxylin–eosin (HE) for histological analysis. The light microscope (Olympus Corp., Tokyo, Japan) was used to evaluate each colon tissue section.

### Enzyme-linked immunosorbent assay (ELISA) analysis

The colon and brain tissues were homogenized and the supernatant was collected. We used commercial ELISA kits and operated according to their instructions. The levels of TNF-α, IL-6, and IFN-γ (Irons Biotechnology Co., Ltd. Huangshi City, Hubei Province, China) in the colon tissue were measured. SP and VIP (Wuhan Cloud-Clone Technology Co., Hubei Province, China) levels in the colon and brain tissues were measured.

### Western blotting

The expression of TJ proteins was detected using Western blotting. Radioimmunoprecipitation assay buffer (RIPA) (Beyotime Biotechnology, China) was used to extract the total protein. The BCA kit was used to measure the total protein content (KGI Biological Development Co., Ltd., Nanjing, China). Sulfate-polyacrylamide gel electrophoresis (SDS–PAGE) was used to separate proteins, and then they were transferred to polyvinylidene fluoride (PVDF) membranes (IPVH00010, Millipore, Bedford, MA, USA). The primary anti-ZO-1 (No. ab216880, Abcam, Cambs, UK), anti-claudin-1 (No. sc-166338, Santa Cruz Biotechnology, USA) and anti-occludin (No. ab216327, Abcam, Cambs, UK) antibodies were used to incubated overnight at 4 °C.After washing with TBST, the membranes were incubated with HRP-coupled secondary antibodies (southern Biotech, USA) for 2 h at 4 °C. The StarSignal system (GenStar, Beijing, China) was used to identify protein signals, and Image J image analysis software was used to analyze the grayscale values of the target strips.

### 16S rRNA gene sequencing of the gut microbiota

Samples of the feces and caecum contents were taken and immediately frozen at −80 °C for subsequent analysis. Genomic DNA was isolated from the samples, and the 16S rRNA V3–V4 region was sequenced using DNA pyrophosphate. The PCR products were purified and homogenized to equal concentrations. The products were then subjected to paired-end (PE) library construction and sequencing on the Illumina MiSeq platform (Illumina, USA). Following sample differentiation, operational taxonomic units (OTU) clustering analysis was performed using the PE reads produced from MiSeq sequencing. The PE reads were first spliced by the overlap relationship, while the sequence quality was quality-controlled and filtered. OTU clustering based on 97% similarity to nonrepeating sequences (excluding single sequences) was carried out using the software platform Uparse (version 7.0.1090 http://drive5.com/uparse/), and OTU representative sequences were obtained using Ribosomal Database Project (RDP) Classifier (version 2.11 http://sourceforge.net/projects/rdp-classifier/) for taxonomic analysis.

### Statistical analysis

The data were analyzed using GraphPad Prism 8 software, and the outcomes were expressed as the mean ± standard error of mean (SEM). In processing the data from ELISA and Western blotting, the findings were compared between the six groups using one-way ANOVA. The Tukey method was used for multiple comparisons when the groups had the same number of members, otherwise, we chose Bonferroni method. To investigate the variations in bacterial composition between the two groups, the Wilcoxon rank-sum test was applied. The difference is shown to be statistically significant at *p*** ***<*** **0.05.

## Results

### HPLC analysis of SNS

The contents of saikosaponin A, paeoniflorin, naringin, and glycyrrhizic acid were detected by HPLC. There were 0.109 mg/mL saikosaponin A, 4.483 mg/mL paeoniflorin, and 4.261 mg/mL glycyrrhizic acid (shown in Table 2).

**Table 2.** HPLC analysis results of SNS.

Compound name	Molecular formula	Reference standards RT (min)	SNS sample RT (min)	Amount (mg/mL)
Saikosaponin A	C_42_H_68_O_13_	67.300	67.287	0.109
Paeoniflorin	C_23_H_28_O_11_	24.557	24.534	4.483
Naringin	C_27_H_32_O_14_	33.883	34.947	Undetected
Glycyrrhizic acid	C_42_H_62_O_16_	63.594	63.634	4.261

### Effects of SNS on colonic tissue morphology in WIRS mice

**Figure 1.**
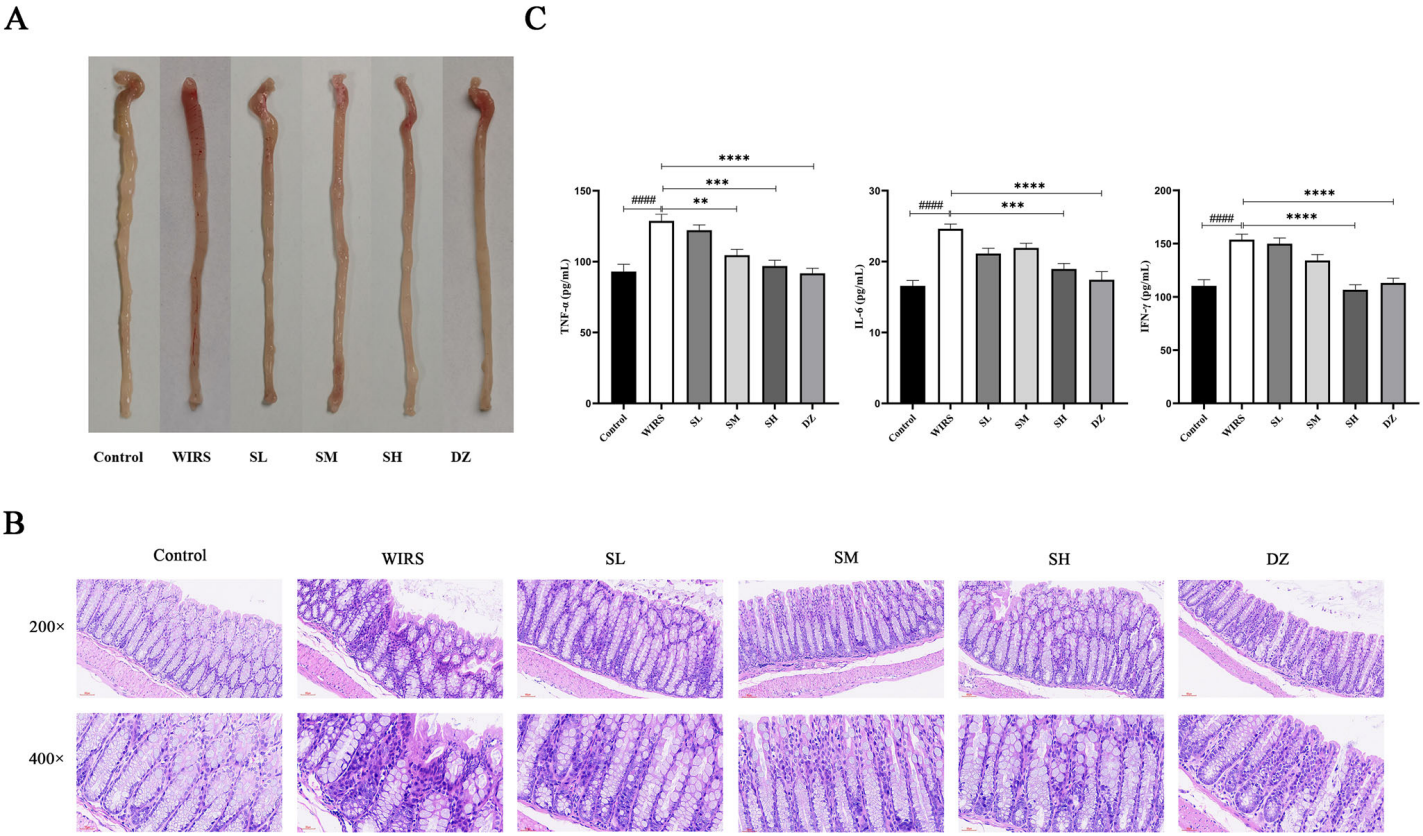
Effects of SNS on colon histopathological changes in WIRS mice. (A) Photographs of colonic tissue from 6 groups of mice. (B) Histopathological examination of 6 groups of mice colonic tissue. (C) Effects of SNS on the levels of TNF-α, IFN-γ and IL-6 in colon tissue of mice in 6 groups (*n* = 6-8). (^####^*p <* 0.0001 vs. the control group; **p < 0.01, ***p < 0.001, ****p < 0.0001 vs. the WIRS group). Control: control group, WIRS: water immersion restraint stress group, SL: SNS low-dose group, SM: SNS middle-dose group, SH: SNS high-dose group, DZ: diazepam group.

Through visual observation of the colonic tissue in each group, it was found that capillaries were visible in the control group with no hemorrhage or reddening. Diffuse bleeding and redness were observed on the surface of the colon in the WIRS group. In contrast, the degree of colonic hemorrhage in the SL, SH, and DZ groups showed visual improvement in the WIRS group (shown in (Figure 1(A)).

HE staining (Figure 1(B)) showed that the colon tissue structure of the control mice was clear, without edema or congestion, the mucosal epithelial cells were orderly arranged, the surface was smooth, the intestinal glands were normal, and there was no inflammatory cell infiltration. The WIRS mice had localized loss of colonic mucosal epithelium, wrinkled and detached, disturbed glandular structure, reduced cup cells, and inflammatory cell infiltration in the mucosal layer. Compared with the WIRS group, no edema was seen in the submucosa of the colon in the three groups of SNS pretreatment, lymphoid tissue was abundant, and inflammatory cell infiltration was reduced. Inflammatory cell infiltration increased in the SM group compared to the SL group. However, there was no inflammatory cell aggregation in the basal layer of the SH group. In comparison to the WIRS group, the DZ group had only a minor degree of inflammatory cell infiltration of the colon mucosa.

### Effects of SNS on the levels of TNF-α, IL-6, and IFN-γ in the colonic tissue of mice in WIRS mice

TNF-α, IFN-γ, and IL-6 are often used as key inflammatory markers in the assessment of colonic inflammation. As shown in Figure 1(C), TNF-α, IL-6, and IFN-γ levels in the WIRS group were considerably higher than those in the control group (*p*** ***<*** **0.0001). Compared to that in the WIRS group, the TNF-α level was markedly reduced in the SM group (*p*** ***<*** **0.01); TNF-α, IL-6, and IFN-γ levels were lowered in the SH (*p*** ***<*** **0.001 or *p*** ***<*** **0.0001) and DZ (*p*** ***<*** **0.0001) groups.

### Effects of SNS on the levels of SP and VIP in the colon and brain tissues of WIRS mice

**Figure 2.**
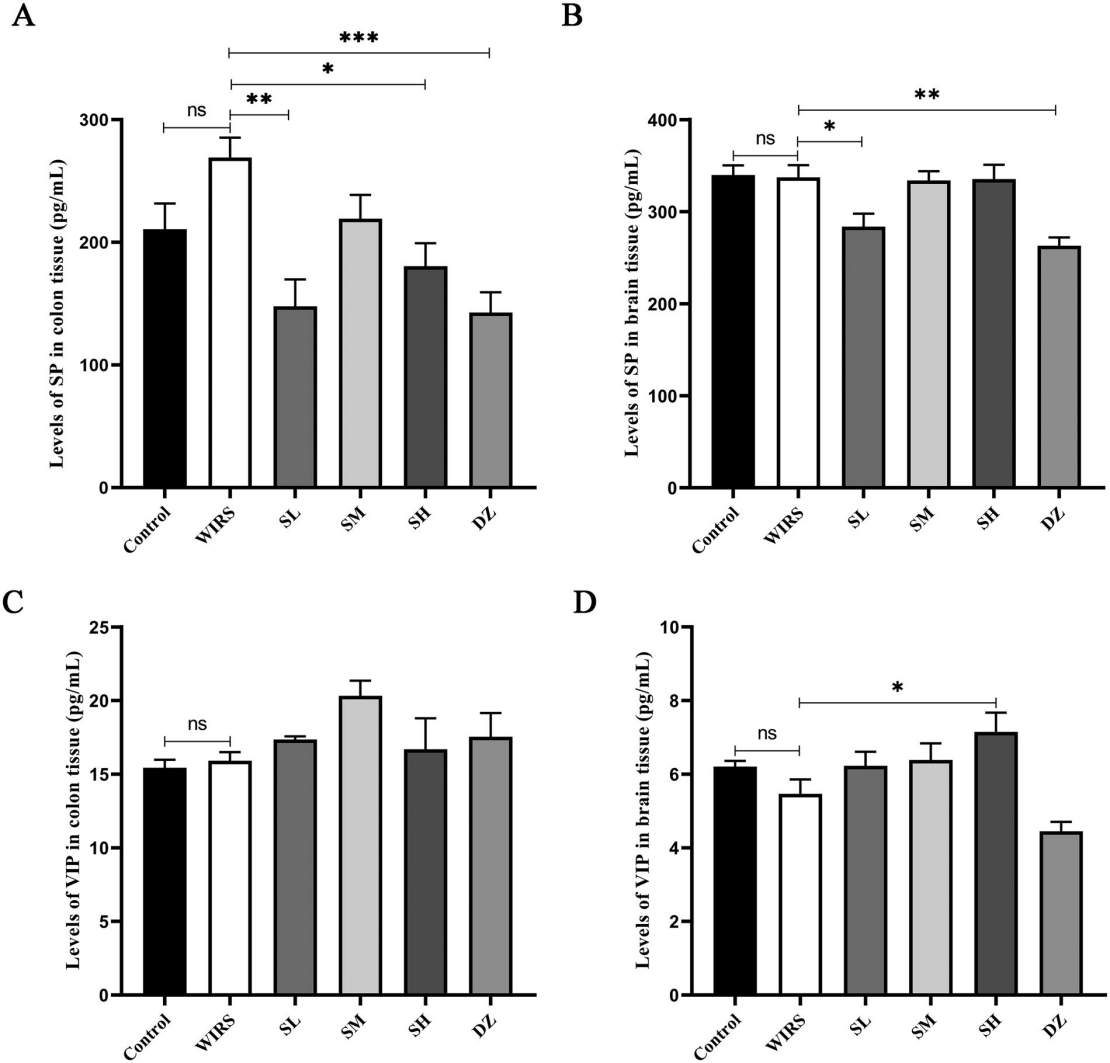
Effects of SNS on the levels of SP and VIP in the colon and brain of mice in 6 groups (*n* = 6). (*p < 0.05, **p < 0.01, ***p < 0.001 vs. the WIRS group).

When compared to the control group, the levels of SP and VIP in the colon and brain tissues were not significantly different in the WIRS group (*p*** ***>*** **0.05) (Figure 2). Although the WIRS group had higher levels of SP in the colonic mucosa, this increase was not statistically significant. With drug pretreatment, there was a statistically significant difference between the colon tissue SP levels in the SL, SH, and DZ groups and the WIRS group, the SL, SH, and DZ groups had lower levels (*p*** ***<*** **0.05, *p*** ***<*** **0.01, or *p*** ***<*** **0.001). When compared to the control group and WIRS group, the SP level in brain tissue was significantly lower in the SL and DZ groups (*p*** ***<*** **0.05 or *p*** ***<*** **0.01). Compared to the WIRS group, VIP level in brain tissue was significantly higher in the SH group (*p*** ***<*** **0.05), and was significantly lower in the DZ group compared to the control group (*p*** ***<*** **0.05).

### Effects of SNS on the protein expression of ZO-1, claudin-1, and occludin in WIRS mice

**Figure 3.**
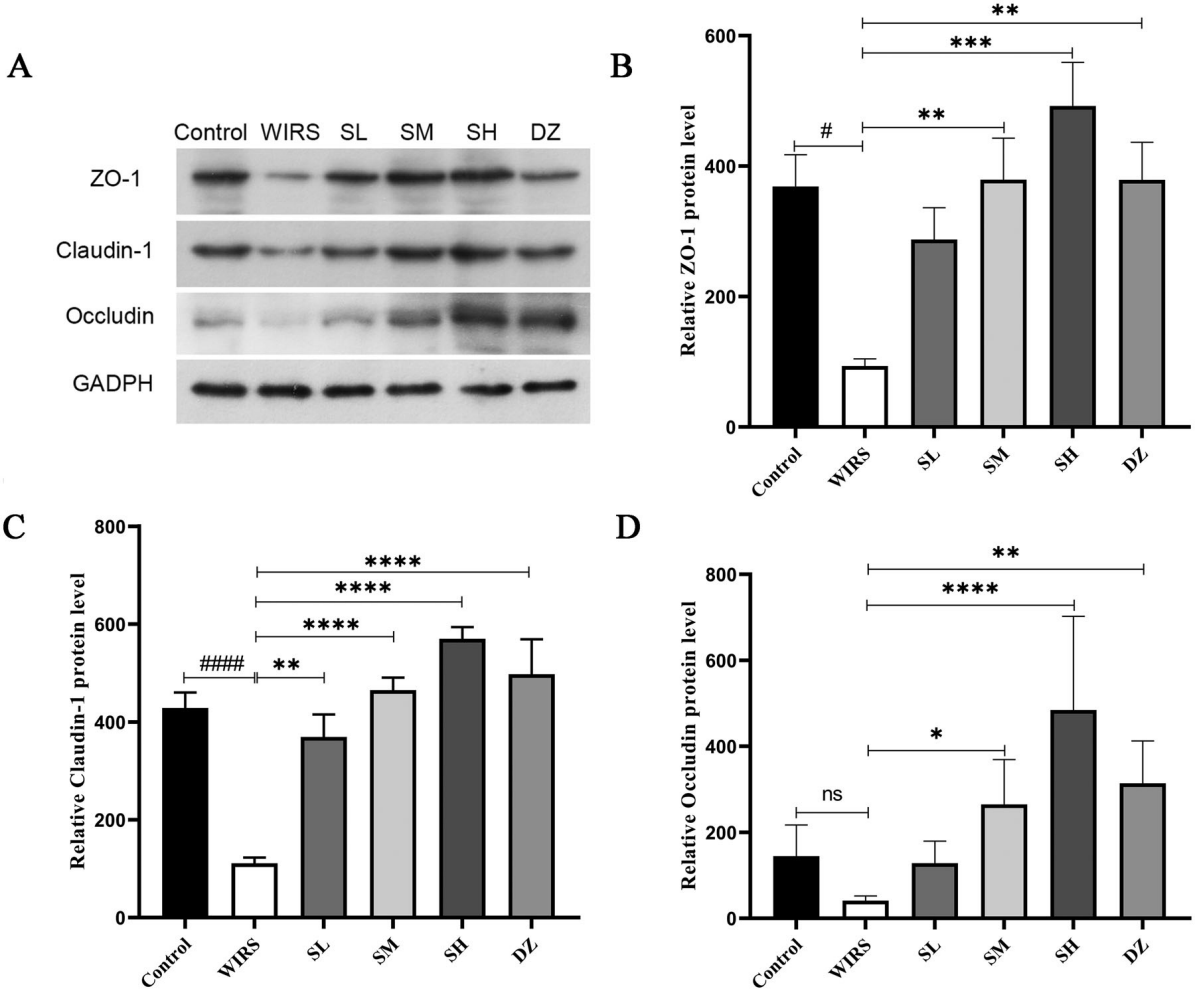
Effects of SNS on the protein expression of ZO-1, claudin-1, and occludin in the colon tissue of mice in 6 groups. (^#^*p <* 0.05, *^####^p <* 0.0001 vs. the control group. *p < 0.05, **p < 0.01, ***p < 0.001, ****p < 0.001 vs. the WIRS group).

To determine whether SNS affected the level of TJ expression in colon tissue, we performed Western blotting. According to the data (Figure 3), the WIRS group had lower levels of ZO-1 and claudin-1 (*p*** ***<*** **0.05 or *p*** ***<*** **0.0001), compared to the control group. There was also a nonsignificant decrease in occludin expression (*p*** ***>*** **0.05). The expression of the claudin-1 protein was increased in the SL group compared with that in the WIRS group (*p*** ***<*** **0.01), and ZO-1, claudin-1, and occludin were also increased in the SM, SH and DZ groups (all *p*** ***<*** **0.05). These findings implied that SNS may improve intestinal permeability by controlling the expression of TJ proteins, and the best outcome was found in the SH group.

### The impact of SNS on the intestinal flora in WIRS mice

#### Bacterial populations and gut diversity and richness

**Figure 4.**
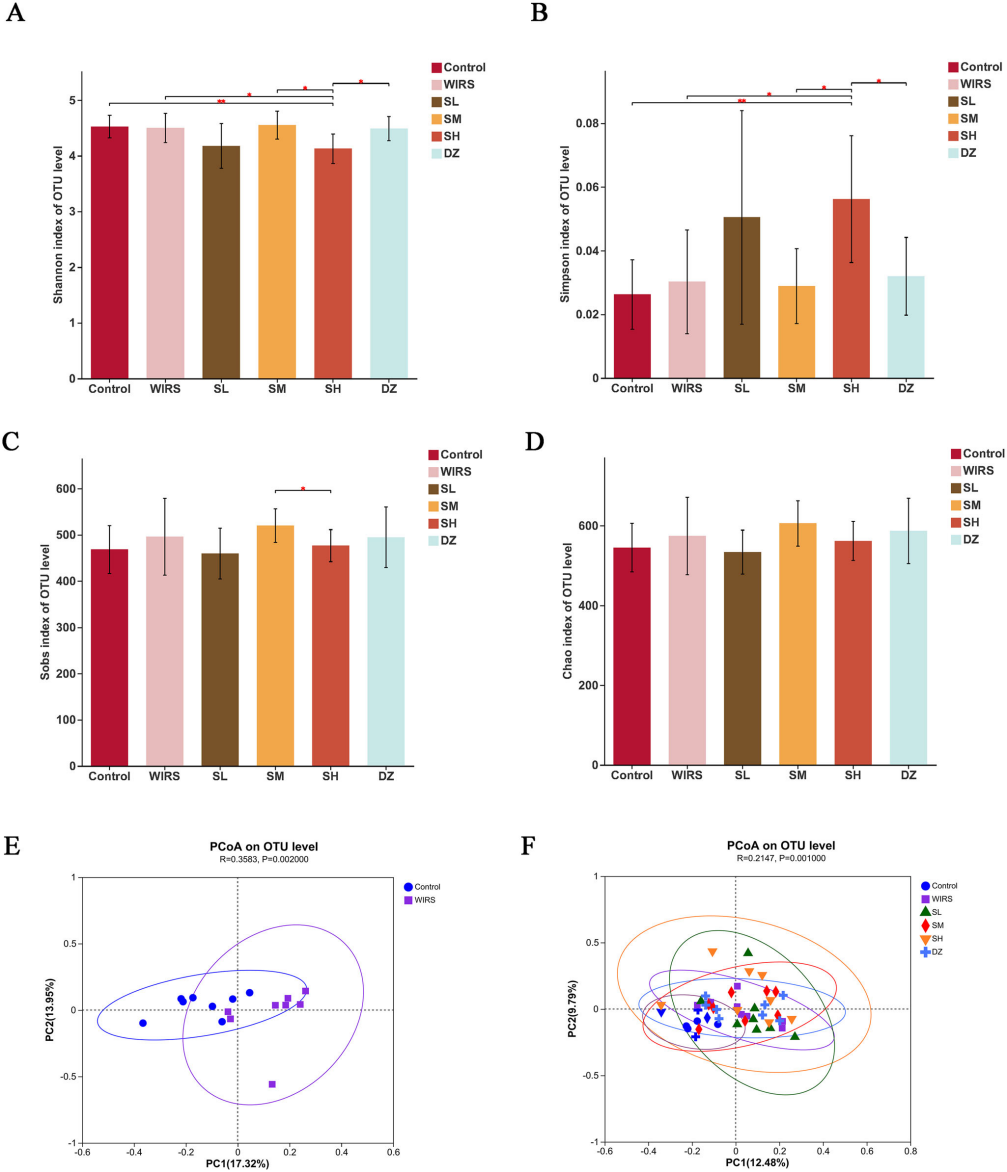
Bacterial populations in the gut diversity and richness. (A-D) Alpha diversity of the gut microbiota between the 6 groups (*p < 0.05, **p < 0.01). (E) PCoA based on OTU table of the control group and the WIRS group. (F) PCoA based on OTU table of the 6 groups.

Forty-eight samples were sequenced, and 3183351 optimal sequences were obtained, with an average sequence length of 414 bp. Alpha diversity is an ecological indicator of the number and distribution uniformity of taxons in each sample. The alpha diversity of the WIRS group was not significantly different from that of the control group, including Shannon’s index, Simpson’s index, Sobs’ index, and Chao’s index (Figure 4(A–D)).

Beta diversity is an index to compare the community composition microbiome and evaluate the differences between microbiota. In this study, we used Principal co-ordinates analysis (PCoA) to determine beta diversity in each group. The PCoA showed contributions of 12.48% for PC1 and 9.79% for PC2, with different distribution densities and spatial distances between the groups (Figure 4(F)). According to the Bray-Curtis distance at the OTU level (*R* = 0.3583, *p* = 0.002), the control group was isolated from the WIRS group (Figure 4(E)), demonstrating changes in gut flora in comparison to the control group.

#### Composition differences between groups at the phylum and genus levels

**Figure 5.**
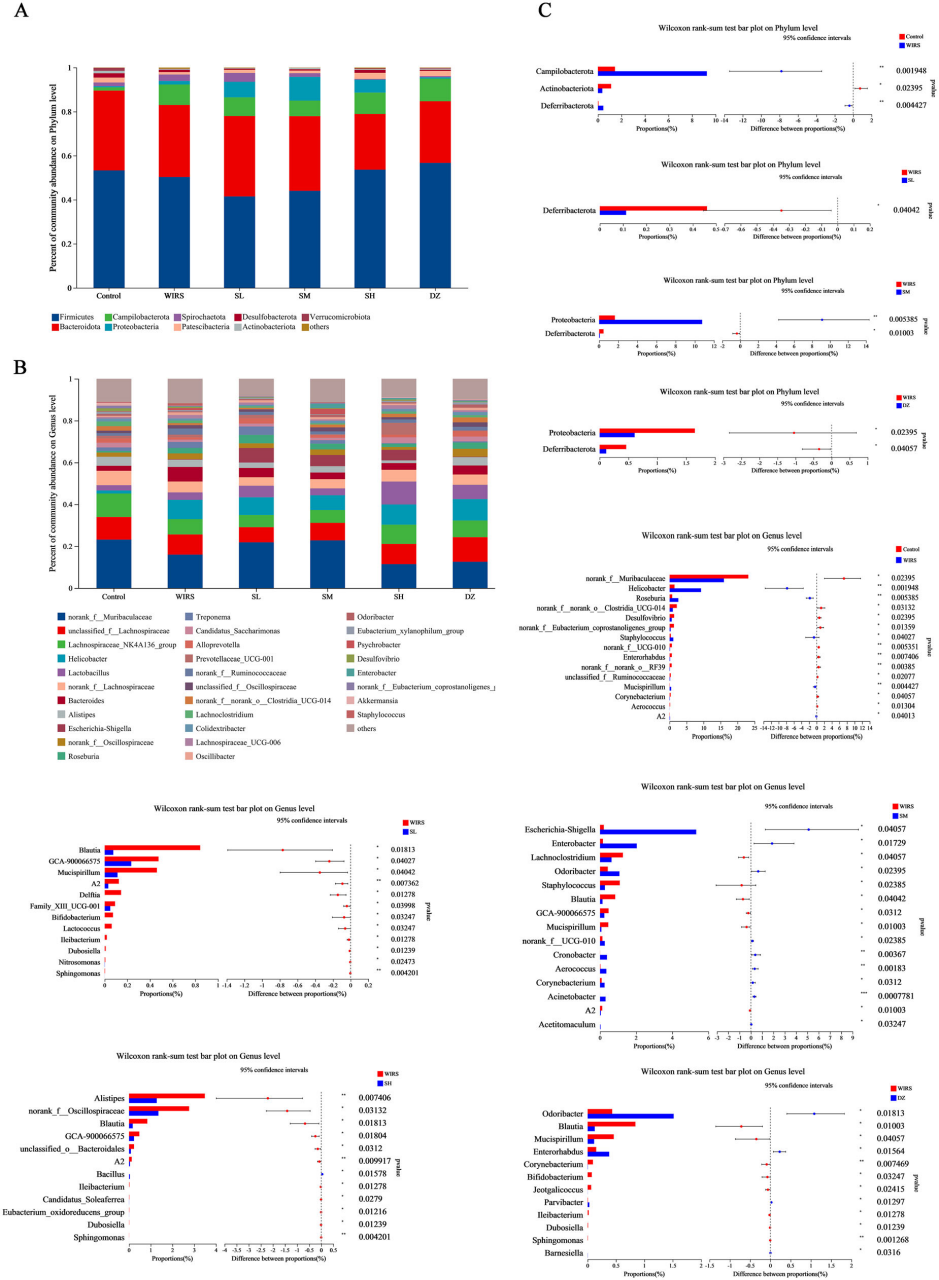
Disparities in group composition at the phylum and genus levels. (A) The assessment of each group’s phylum level abundance of the gut microbiota composition. (B) The assessment of each group’s gut microbiota abundance at the genus level. (C) Differences in intestinal flora at the phylum and genus levels.

At the phylum level, nine major bacterial phyla were discovered (Figure 5(A)), including *Firmicutes*, *Bacteroidetes*, Campilobacterota, *Proteobacteria*, *Spirochaetota*, *Patescibacteria*, *Desulfobacteria*, *Actinobacteria*, and *Verrucomicrobiota*, with two groups of probiotic bacteria, *Firmicutes* and *Bacteroidetes*, as the dominant groups in each group. As shown in Figure 5(C), the proportion of the Campilobacterota and *Deferribacterota* phyla increased significantly, and that of *Actinobacteriota* decreased significantly (*p* < 0.05). Compared to the WIRS group, the relative Deferribacterota abundance was considerably decreased in the SL and SM groups (*p* < 0.05), *Proteobacteria* was increased in the SM group (*p* < 0.05), and *Proteobacteria*, *Deferribacterota* were significantly decreased in the DZ group (*p*** ***<*** **0.05). There was no statistically significant difference between the WIRS group and the SH group.

At the genus level, each group had 31 dominant bacterial populations (Figure 5(B)). The relative abundances of norank_f_Muribaculaceae, norank_f__norank_o__Clostridia_UCG-014, *Desulfovibrio*, and *norank_f__Eubacterium_coprostanoligenes_group*, etc., decreased in the WIRS group (*p*** ***<*** **0.05), while the abundances of *Helicobacter*, *Roseburia*, *Staphylococcus* and *Mucispirillum*, etc., increased (*p*** ***<*** **0.05) compared to the control group. Compared with the WIRS group, *Blautia* and GCA-900066575 were significantly reduced in the SL, SM, and SH groups. The abundance of *Mucispirillum* decreased significantly in the SL, SM, and DZ groups (*p*** ***<*** **0.05); the *Escherichia-Shigella*, *Enterobacter*, and *Odoribacter* abundances significantly increased (*p*** ***<*** **0.05) in the SM group, and those of *Lachnoclostridium* and *Staphylococcus* significantly decreased (*p*** ***<*** **0.05); *Alistipes* and *norank_f__Oscillospiraceae* abundances decreased (*p*** ***<*** **0.05) in the SH group; and *Enterobacter* and *Odoribacter* abundances considerably increased (*p*** ***<*** **0.05) in the DZ group (Figure 5(C)).

#### Identifying the bacterial taxa with the most distinctive characteristics

**Figure 6.**
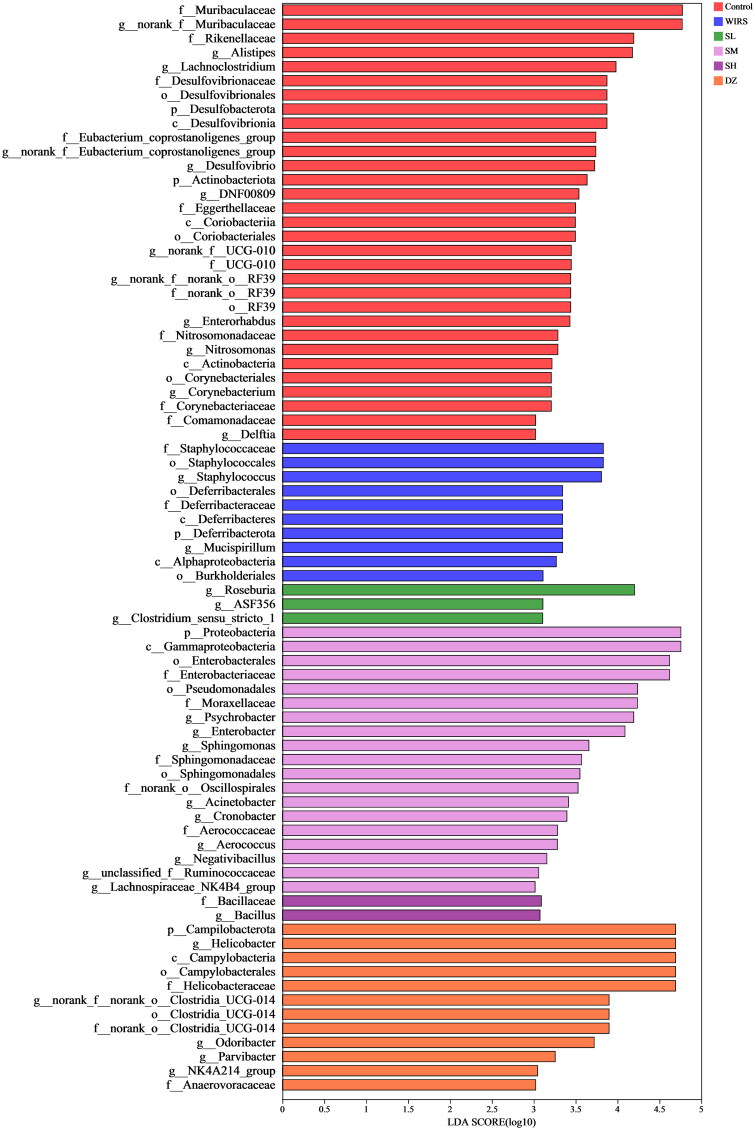
LEfSe (LDA score>3.0) between 6 groups, from the phylum level to the genus level.

Linear discriminant analysis (LDA) Effect Size (LEfSe) showed that mice in the WIRS group were enriched with f__*Staphylococcaceae*, o__Staphylococcales, g__*Staphylococcus*, g__*Mucispirillum*, p__Deferribacterota, o__Deferribacterales, c__Deferribacteres, f__*Deferribacteraceae*, c__Alphaproteobacteria, o__Burkholderiales, which can be used as biomarkers of WIRS (Figure 6). However, these bacteria were less enriched in mice pretreated with SNS, the abundance of Deferribacterota was significantly lower in the SL and SM groups at the phylum level, the *Staphylococcus* abundance was significantly lower in the SM group at the genus level, and the Mucispirillum abundance was significantly lower in the SL and SM groups (Figure 5(C)).

#### Correlation of intestinal flora changes with inflammatory factors and TJ protein parameters

**Figure 7.**
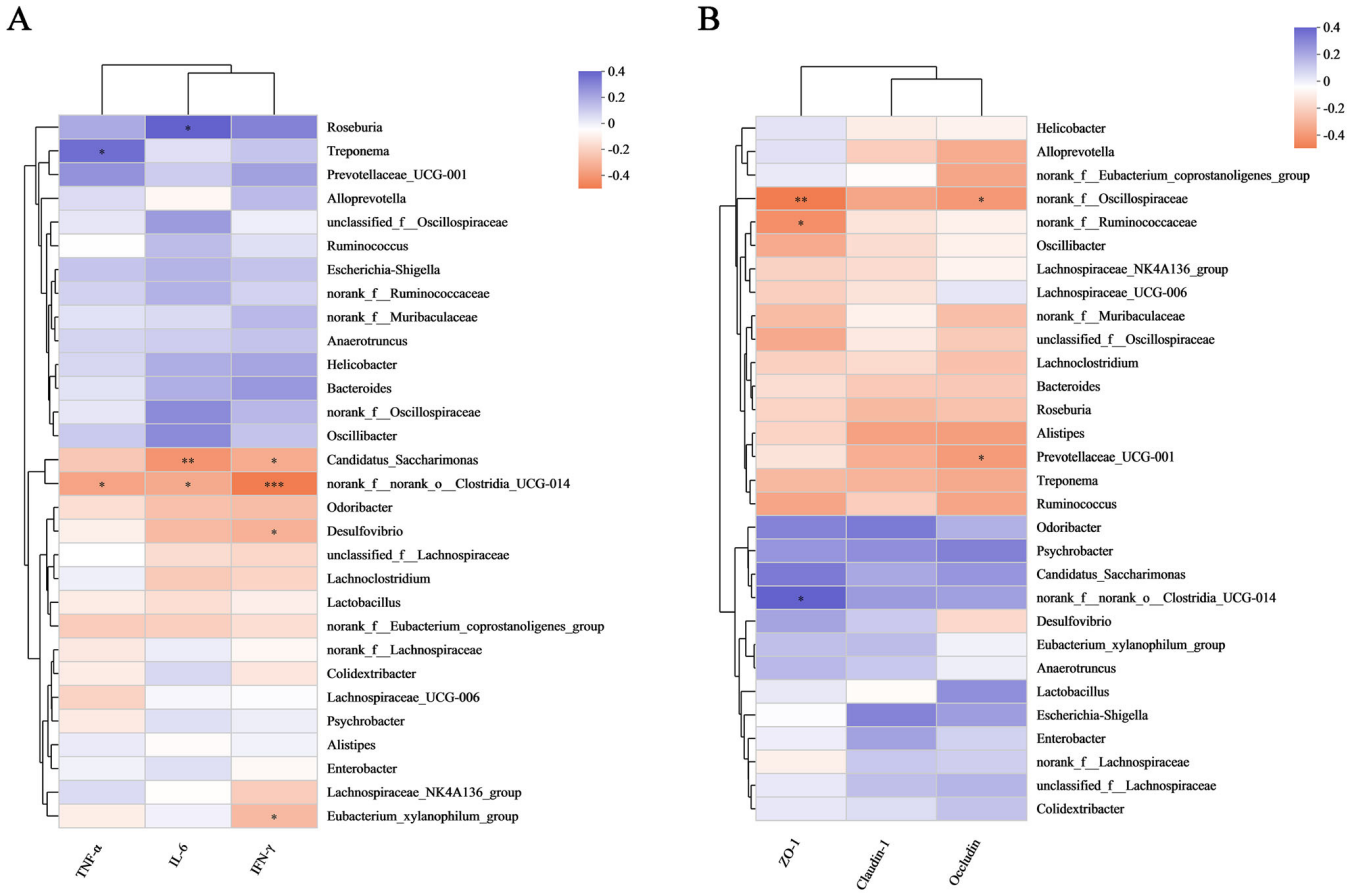
Correlation of intestinal flora changes with inflammatory factors and TJ protein parameters. (A) Analysis of relationships between species abundances and TNF-α, IL-6, and IFN-γ levels. (B) Analysis of relationships between species abundances and ZO-1, claudin-1 and occludin levels. The data in the two-dimensional matrix is reflected by the color change. The size of the value is represented by the color depth, and the change is represented by the color gradient. Orange, which denotes a negative correlation, and purple, which shows a positive correlation, frequently appear as regions on the graph. The absolute value of the correlation coefficient increases as the hue becomes deeper (*p < 0.05, **p < 0.01 and ***p < 0.001).

We also analyzed the role of these bacteria in inflammation (Figure 7(A)). *Roseburia* abundance was positively correlated with IL-6 levels (*p*** ***<*** **0.05); *Treponema* abundance was positively correlated with TNF-α levels (*p*** ***<*** **0.05); norank_f__norank_o__Clostridia_UCG-014 abundance was negatively correlated with TNF-α (*p*** ***<*** **0.05), IL-6 (*p*** ***<*** **0.05), and IFN-γ (*p*** ***<*** **0.001) levels; and *Desulfovibrio* and *Eubacterium*_*xylanophilum_group* abundances were negatively correlated with IFN-γ levels (*p*** ***<*** **0.05). As shown in Figure 7(B), *norank_f__Oscillospiraceae* abundance was negatively correlated with ZO-1 (*p*** ***<*** **0.01) and occludin (*p*** ***<*** **0.05) levels, *Prevotellaceae_UCG-001* abundance was negatively correlated with occludin level (*p*** ***<*** **0.05), and norank_f__*Ruminococcaceae* and norank_f__norank_o__Clostridia_UCG-014 abundances were negatively correlated with ZO-1 levels (*p*** ***<*** **0.05).

## Discussion

Acute psychological stress increases human intestinal permeability (Vanuytsel et al. 2014). The expression of ZO-1 and claudin-1 was significantly reduced in the colonic tissue of WIRS mice (Figure 3), indicating that intestinal barrier function was impaired in WIRS mice. Stress-induced barrier defects promote the cross-cell absorption of colonic mucosa, which enable the absorption of immunogenic chemicals and cause or aggravate intestinal inflammation (Saunders et al. 2002). It is helpful to repair the intestinal barrier by improving the expression and structure of TJ proteins (Choi et al. 2016). Restraint stress aggravates the degree of colonic mucosal damage in experimental colitis mice (Schultz et al. 2015). TNF-α, IFN-γ, and IL-6 are often used as inflammatory markers to assess the degree of colonic inflammation (Rosenstiel et al. 2003; Wu et al. 2021). Our study results demonstrate that SNS and diazepam pretreatment effectively reduced TNF-α, IL-6, and IFN-γ levels in WIRS mice and increased the expression of TJ proteins such as claudin-1, occludin, and ZO-1. These effects were helpful to mitigate the damage caused by WIRS in mice. Both SNS and diazepam were effective in improving colonic inflammation and maintaining colonic mucosal barrier function. Additionally, the effects of the SH group were better than the SL and SM groups.

The brain-gut axis is a neuro-endocrine network that connects information from the emotional and cognitive centers of the brain to peripheral gut function (Khlevner et al. 2018). When the brain-gut axis is stimulated by various stresses, neuropeptides are released (Stasi et al. 2012). However, our study found that there were no significant changes in neuropeptide SP and VIP levels in brain and colonic tissues of WIRS mice. It may be related to the short half-life of neuropeptides in tissues (Mashaghi et al. 2016). The gut flora plays a significant role in the development of many diseases, including autism, mood disorders (Mangiola et al. 2016), IBD (Lloyd-Price et al. 2019), and functional gastrointestinal disorders (Labanski et al. 2020). 16S rRNA gene sequencing was used to observe gut microbiota. We found differences in the β-diversity (PCoA) of the microbial communities between the WIRS and control groups. After WIRS, we observed a considerable increase in the abundances of the phyla Campilobacterota and Deferribacterota, as well as a large decrease in the abundance of Actinobacteriota (Figure 5), compared to the control group. *Campylobacter jejuni* is a widespread foodborne bacterial pathogen that has been shown to promote intestinal inflammation (He et al. 2019). The decline of *Actinobacteriota* abundance was also seen in the UC model (Hua et al. 2021). The abundance of *Deferribacterota* has been previously observed to increase in many experimental animal models, such as the depressive-like behavior model (Sun et al. 2023), diarrhea-predominant IBS model (Li et al. 2020), IBD model (Munyaka et al. 2016; Jian et al. 2021), and colitis-associated carcinoma (CAC) model (Gobert et al. 2022). Compared to the WIRS group, the abundance of *Deferribacterota* decreased significantly in the SL, SM and DZ groups. At the genus level, the relative abundances of *Staphylococcus*, *Mucispirillum* and A2 were significantly increased in the WIRS group than in the control group, whereas the *Staphylococcus* abundance was reduced in SM group, the *Mucispirillum* abundance was reduced in the SL, SM and DZ groups, and the A2 abundance was reduced in the SL, SM and SH groups, compared to the WIRS group. *Staphylococcus* is a recognized pathogenic bacterium for diarrhea and intestinal inflammation, and it is mostly increased in patients with digestive system diseases and colitis models (Li et al. 2021). *Mucispirillum* is associated with mood disorders and has also been found to be related to inflammation of the gut caused by "leakage" of the gut (M. Zhang et al. 2021). *Deferribacterota*, *Staphylococcus*, *Mucispirillum* and A2 may be the key bacteria for SNS to improve the colon injury caused by WIRS.

Moreover, we discovered that WIRS decreased the abundances of norank_f_Muribaculaceae, norank_f_ norank_o_Clostridia_UCG-014, and *Desulfovibrio* and increased the abundances of *Helicobacter* and *Roseburia* (Figure 5(D)) at the genus level. The increase in norank_f_Muribaculaceae and norank_f_norank_o_Clostridia_UCG-014 abundances was beneficial to UC mice. Norank_f_norank_o_Clostridia_UCG-014 promotes advantageous bacteria, inhibits harmful intestinal, and increases nutritional absorption, digestion, and assimilation (Y. Liu et al. 2021; Wang et al. 2021). As shown in Figure 7, there was a negative correlation between norank_f__norank_o__Clostridia_UCG-014 abundance and TNF-α, IL-6, and IFN-γ levels. *Desulfovibrio* spp. is gram-negative bacteria, most of whose members are lipopolysaccharide (LPS) producers, disrupting the intestinal barrier (Zhang et al. 2018). *Desulfovibrio* was widely distributed in the control group, but it was less in the WIRS group, according to LEfSe analysis (Figure 6). A negative correlation between *Desulfovibrio* abundance and the level of IFN-γ (Figure 7A), suggesting that *Desulfovibrio* may play a beneficial role. *Roseburia* has an anti-DSS-induced colitis effect (Wu et al. 2020). However, our study showed a positive association between *Roseburia* abundance and IL-6 levels.

We discovered that the abundances of *Blautia* and GCA-900066575 decreased with the three SNS doses. The more abundance of *Blautia* was found in the gut microbiota of UC and IBS patients than in healthy people (X. Liu et al. 2021). *Odoribacter splanchnicus* can protect mice from colitis and colorectal cancer (Xing et al. 2021). When compared to the WIRS group, the SM group had a considerably lower level of *Lachnoclostridium*. The *Lactobacillus* content in the fecal flora of UC carcinogenesis model mice was low, and the *Lachnoclostridium* content was high (Wang et al. 2018). The SM group regulated the balance of intestinal flora by increasing the abundance of the probiotic *Odoribacter* and reducing the abundances of pathogenic bacteria such as *Lachnoclostridium* and *Staphylococcus. Alistipes* and *Odoribacter* were negatively correlated with inflammatory cytokines (Guo et al. 2021). The increase in the abundance of *norank_f__Oscillospiraceae* may exacerbate the symptoms of rheumatoid arthritis (Hu et al. 2022). The abundances of *Alistipes* and *norank_f__Oscillospiraceae* were significantly reduced in the SH group. Our research revealed a negative correlation between *norank_f__Oscillospiraceae* abundance and ZO-1, occludin levels, suggesting that the SH group may enhance intestinal barrier function by reducing the abundance of *norank_f__Oscillospiraceae.* In the Spearman correlation analysis of intestinal flora (Figure 7), it was found that the alterations of inflammatory factors and TJ protein parameters in WIRS mice were positively and negatively correlated with intestinal flora, which indicated that the colonic inflammatory damage and intestinal barrier damage in WIRS mice were closely related to intestinal flora dysbiosis.

Some studies have confirmed that intestinal beneficial flora can improve intestinal TJ proteins (Ait-Belgnaoui et al. 2014; Mu et al. 2017). Alterations in the tight junctions complex can promote the development of colonic inflammation (Tan et al. 2019). Our research has proved that SNS can improve intestinal flora and TJ proteins. Therefore, we speculate that SNS plays a role in mucosal protection by improving intestinal flora and promoting the formation of TJ proteins. Nevertheless, this study has not yet elucidated the mechanism by which WIRS leads to intestinal dysbacteriosis and TJ proteins, which will be the focus of our subsequent study.

## Conclusions

In conclusion, our findings revealed that SNS could effectively reduce WIRS-induced colon injury, relieve pathological damage of the colon, and regulate the expression of the inflammatory factors and the TJ proteins. In addition, the WIRS group had a different gut flora composition than the control group, and SNS pretreatment alleviated intestinal flora dysbiosis and reshaped the gut flora structure. The positive effect of SNS on WIRS could provide a theoretical basis to treat stress-related gastrointestinal disorders.

## Supplementary Material

Supplemental Material

## Data Availability

All data generated or analyzed during this study are included in this article. Further enquiries can be directed to the corresponding author.
