# Supplementary material for: Sinisan ameliorates colonic injury induced by water immersion restraint stress by enhancing intestinal barrier function and the gut microbiota structure
Source: Pharm Biol. 2023 Apr 4;61(1):598–609. doi: 10.1080/13880209.2023.2191643 (PMC10075512; doi:10.1080/13880209.2023.2191643)
Supplement: Supplemental Material [file IPHB_A_2191643_SM5337.docx]

# Supporting Information

The quality control of Sinisan was conducted by high-performance liquid chromatography (HPLC). Representative fingerprint chromatograms of SNS extract were obtained through HPLC-DAD at the wavelength of 210 nm.


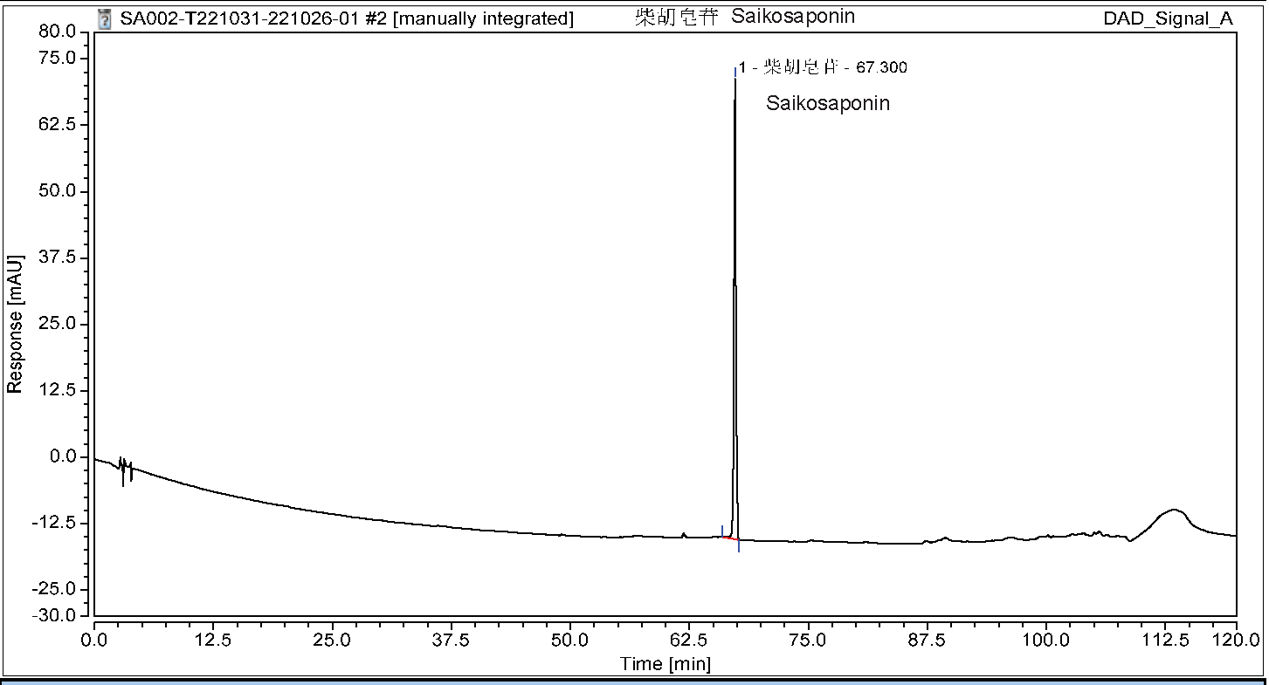


Fig. S1. Representative fingerprint chromatograms of reference standards of saikosaponin A.


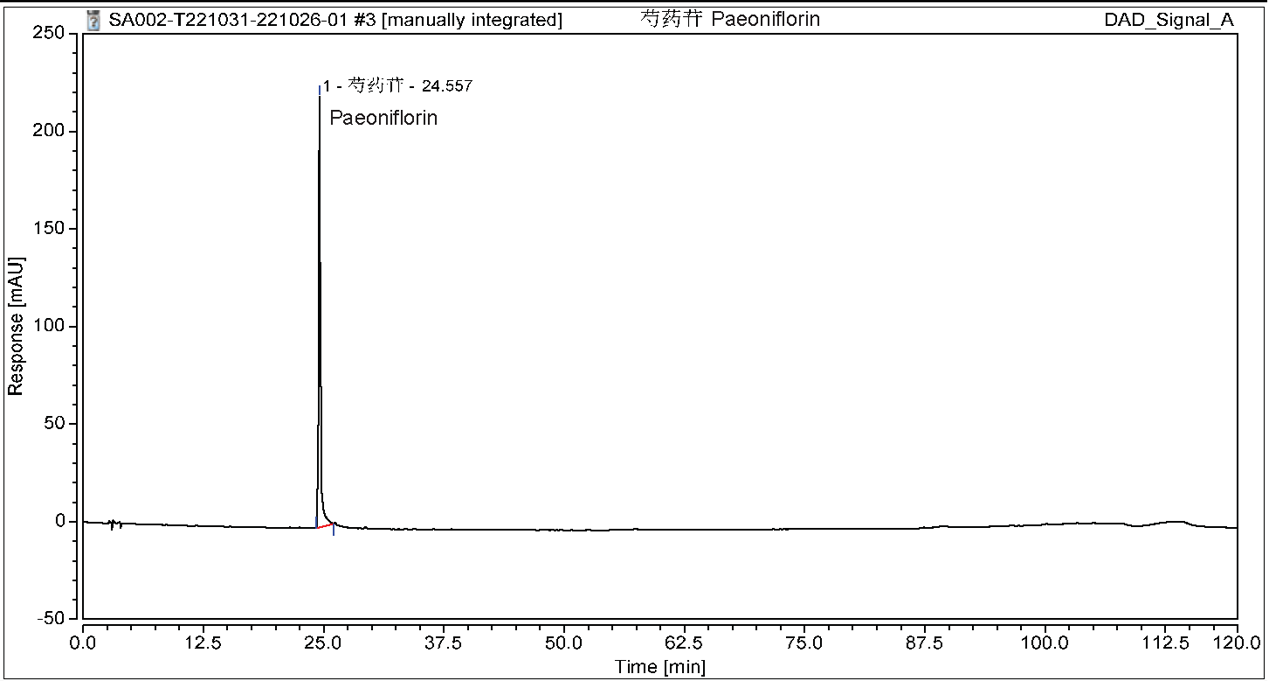


Fig. S2. Representative fingerprint chromatograms of reference standards of paeoniflorin.


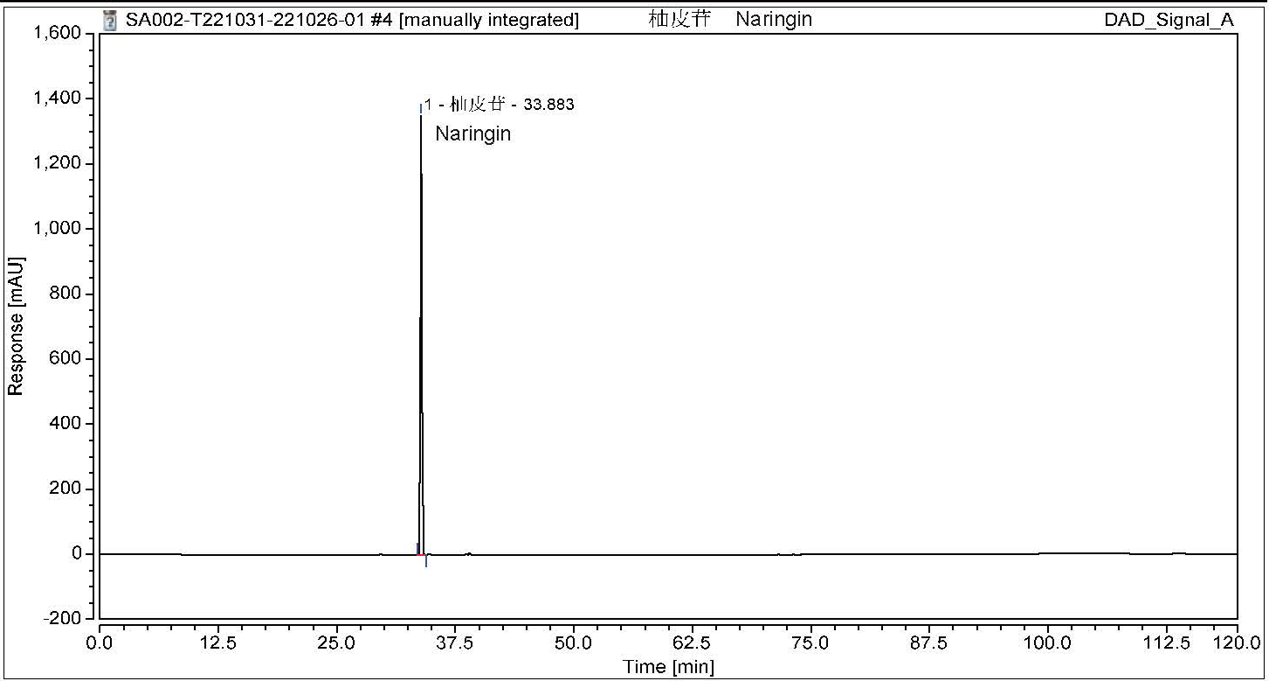


Fig. S3. Representative fingerprint chromatograms of reference standards of naringin.


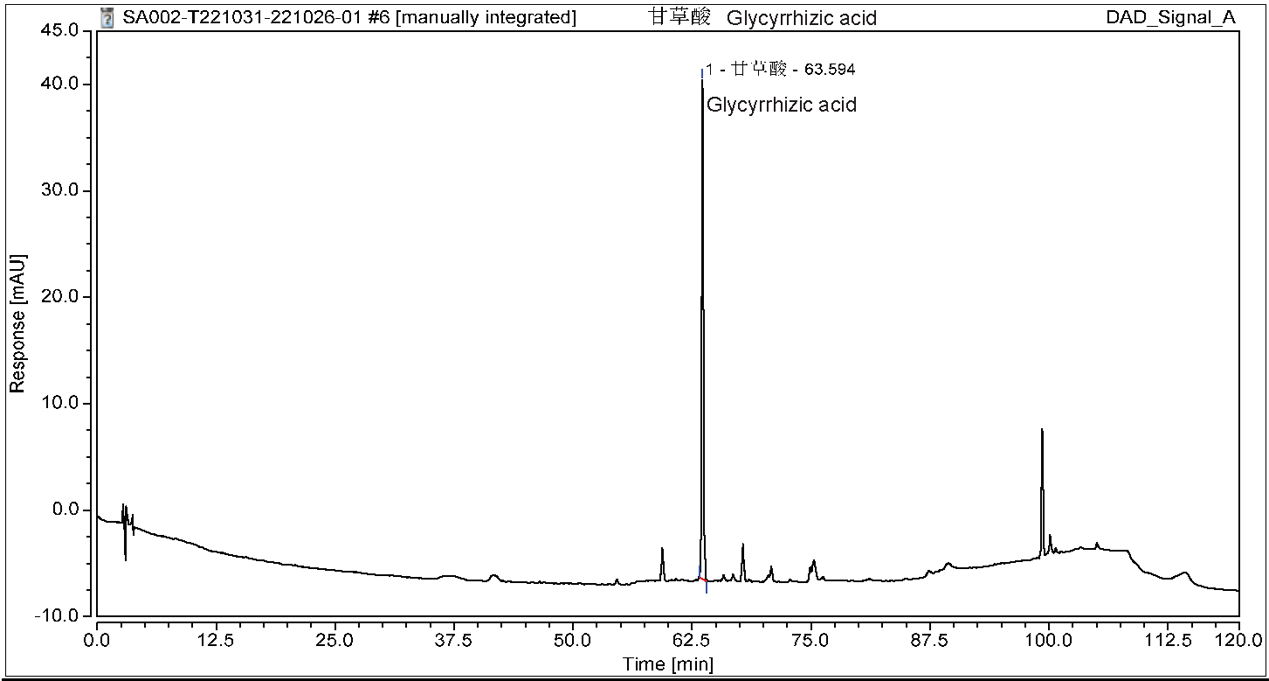


Fig. S4. Representative fingerprint chromatograms of reference standards of glycyrrhizic acid.


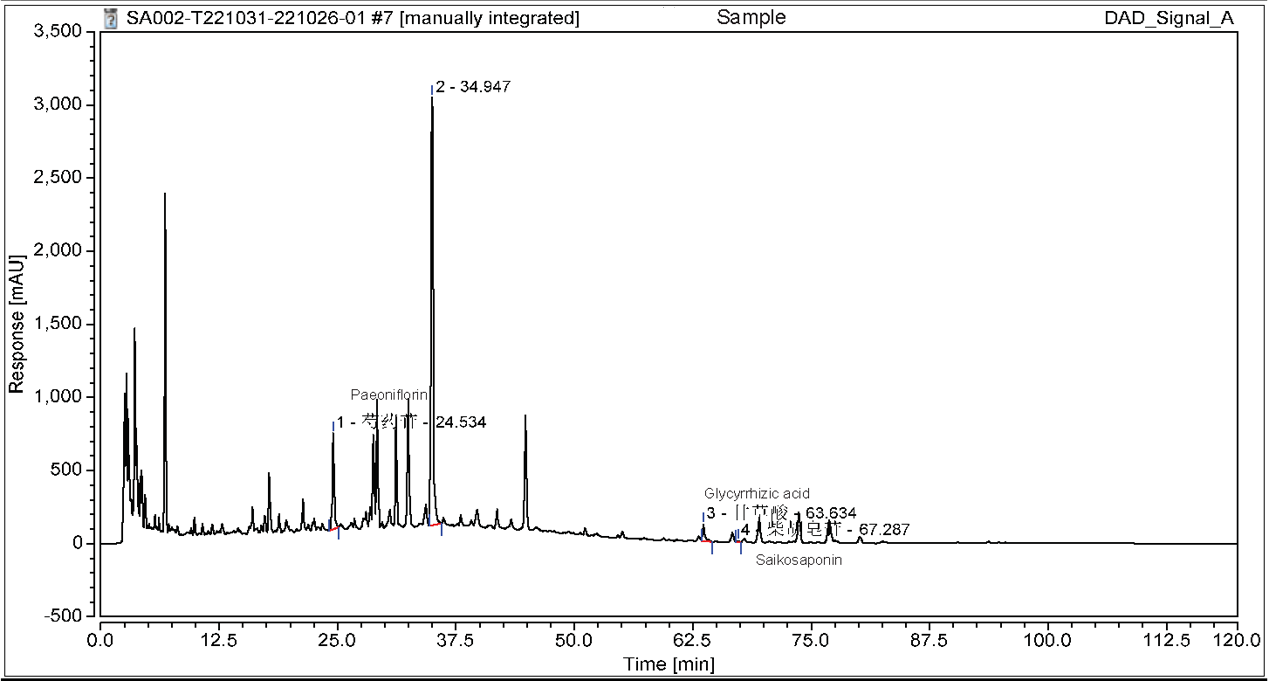


Fig. S5. Representative fingerprint chromatograms of Sinisan sample.

Paeoniflorin (retention time [RT]: 24.534 min), glycyrrhizic acid (RT: 63.364 min), and saikosaponin A (RT: 67.287 min).
